# Supportive care in oral cancer patients: a prospective longitudinal study at a german university hospital

**DOI:** 10.1007/s10006-026-01571-3

**Published:** 2026-05-13

**Authors:** Kröplin Juliane, Reppenhagen Jil-Charlot, Hirsemann Anke, Jan Liese, Bernhard Frerich

**Affiliations:** University Medical Centre Rostock, Schilling Allee 35, Rostock, 18097 Germany

**Keywords:** Perioperative medicine, Outcome quality, Patients’ satisfaction, Immunotherapy, Multimodal therapy, Resilience

## Abstract

**Background:**

Supportive therapeutic interventions aimed at improving outcome quality in oncological patients are key components of perioperative medicine. The present study aims to analyze the use of supportive therapeutic interventions in the pre-, peri-, and postoperative phases in patients with oral cavity carcinomas, as well as the corresponding temporal development of patient-specific resilience and satisfaction.

**Methods:**

This prospective longitudinal study included patients with oral cavity carcinomas who underwent surgery at a university-based oncology center between 2022 and 2025. Patient-specific resilience (RS-11 questionnaire), utilization of supportive therapies (yes/no/irregular), and satisfaction with nine domains of life were assessed. A final survey was conducted four months after surgery (T3) and compared with results from the preoperative (T1) and postoperative inpatient (T2) phases.

**Results:**

A total of 30 patients were enrolled in the study. The average time from diagnosis to treatment was 21 days (max: 35d; min: 9d). The average RS-11 score was 5.7 (T1: 5.3; T2: 4.6). Compared to the preoperative baseline, there was a statistically significant decrease in satisfaction in the domains of physical activity (T1: *p* = 0.01; T2: *p* = 0.53) and enjoyment of food (T1: *p* = 0.01; T2: *p* < 0.001). Three patients received regular psycho-oncological support (T1: *n* = 0; T2: *n* = 23). Seven patients reported regular participation in sports programs (T1: *n* = 2; T2: *n* = 21). Three patients received speech therapy (T1: *n* = 1; T2: *n* = 17).

**Discussion:**

The present results indicate that only a small proportion of patients continue to regularly utilize the supportive therapies initiated in the immediate postoperative period four months after surgery. At the same time, there is a significant decline in satisfaction with physical activity and the ability to enjoy eating when compared to preoperative assessments. Although the greatest needs for supportive therapies were identified during hospitalization, persistent postoperative impairments should be addressed through more consistent use of supportive care in the early outpatient follow-up phase. The structural framework conditions for the use of innovative therapy methods such as perioperative immunotherapy must also be taken into account.

## Introduction

Squamous cell carcinomas of the oral cavity are among the most common malignancies in the head and neck region. Both the presence of these tumours and their treatment are frequently associated with substantial functional and psychosocial burdens for affected individuals [1]. Limitations in eating, speech function, and physical appearance can lead to psychological distress, social isolation, and impairments in emotional processing. These consequences often affect patients’ quality of life far beyond the acute treatment phase [2, 3]. Moreover, emotional and social factors have a significant impact on recovery [3–5].

Supportive measures aim to prevent or alleviate the side effects of cancer and its treatment. At the same time, they are intended to improve patients’ quality of life and the functionality of impaired anatomical structures, without influencing the course of the disease itself [6, 7]. The Multinational Association of Supportive Care in Cancer (MASCC) describes such interventions as an essential component of oncological care. Examples include psycho-oncological services, speech therapy, and exercise or physical activity programs [8]. These interventions can mitigate the consequences of primary treatment, stabilize emotional well-being, and facilitate reintegration into daily life [9]. In particular, multimodal, individually tailored programs delivered during the perioperative phase have already demonstrated positive effects on short- and long-term treatment outcomes in oncologic visceral surgery [10]. Comparable concepts are increasingly being discussed in the management of head and neck tumours [11, 12].

The aim of this prospective longitudinal study is to systematically assess the use of supportive therapeutic interventions during the pre-, peri-, and postoperative phases in patients with oral cavity carcinoma. The study investigates the temporal development of patient-specific resilience as well as satisfaction across various domains of life. Furthermore, the study seeks to determine the extent to which supportive measures are continued after inpatient treatment. The results are intended to help identify existing gaps in care and to highlight approaches for structured outpatient follow-up treatment, as well as potentials for prehabilitation strategies.

## Methods

### Study participants

In this prospective longitudinal study, patients with oral cavity carcinoma who underwent surgery at an oncological centre of a university hospital between 2022 and 2025 were included. All participants provided written informed consent prior to enrolment.

Demographic and clinical baseline data included age, sex, tumour stage according to UICC criteria, and TNM classification.

### Study design and surveys

Data were collected at three defined time points: preoperatively (T1); postoperatively during the inpatient stay (T2; 10th postoperative day); and four months after surgery during outpatient follow-up (T3). At all three time points, the same standardized questionnaires were used to collect general, disease- and therapy-related, as well as satisfaction-related information. The surveys were completed together with the patients to allow for direct support in case of questions regarding comprehension. In addition, the clinically validated RS-11 questionnaire was used to assess individual resilience [13]. The scale ranges from 1 (“does not apply”) to 7 (“applies very much”).

**Fig. 1:**
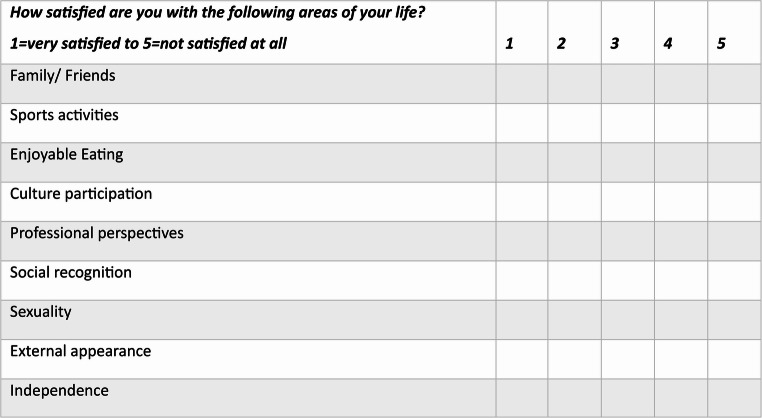
Questionnaire on individual life satisfaction assessed using individually defined items based on Klaus Grawe’s four fundamental psychological needs: attachment, autonomy, pleasure attainment, and self-esteem enhancement [14]. Ratings were provided on a Likert scale ranging from 1 (“very satisfied”) to 5 (“not satisfied at all”)

Overall satisfaction was assessed using individually defined items based on Klaus Grawe’s four fundamental psychological needs: attachment, autonomy, pleasure attainment, and self-esteem enhancement [14]. These nine items were: family and friends, physical activity, participation in cultural activities, professional prospects, sexuality, enjoyable eating, physical appearance, social recognition, and independence. Ratings were provided on a Likert scale ranging from 1 (“very satisfied”) to 5 (“not satisfied at all”); Fig. 1.

The use of supportive therapeutic interventions—such as exercise programs, physiotherapy, psycho-oncological counselling, speech therapy, and self-help groups—was also documented. Participation was recorded using the categories “yes,” “no,” or “irregular”. If only an initial contact had taken place without any further structured follow-up appointments or if only sporadic/unstructured follow-up appointments had taken place, this was considered irregular.

### Data collection and analysis

Statistical analysis was performed using IBM SPSS Version 27. Mean values for resilience and patient satisfaction were analysed using a paired tailed t-test. An open, exploratory analysis without predefined hypotheses was conducted, allowing for the possibility that no significant changes would be observed. A p-value of < 0.05 was considered statistically significant. Exclusion criteria included lack of consent and inability to provide consent. Individuals who developed a recurrence during the postoperative course were excluded from the third survey.

### Ethical statement

All participants were thoroughly informed about the study procedures, processes, and potential risks, and provided written informed consent. The study protocol was reviewed and approved by the responsible ethics committee of the university hospital (A 2022 − 0139).

## Results

### Demographics

A total of 30 patients were enrolled in the study. All patients received primary surgical treatment. Table 1 provides an overview of the patient- and disease-related data collected. With the exception of one adenocarcinoma of the gl. sublingualis, all tumours were classified as squamous cell carcinomas. At time point T3, 27 patients were still available for follow-up. One patient died before the third survey could be completed, one developed an early recurrence, and one did not participate within the required time frame for reasons unknown to us.

**Table 1:** Analysis of demographics (age, gender), nicotin abuse and desease related data (UICC-classification, TNM-Stage, adjuvant radio(chemo)therapie), stated in numbers (n = x) and persentages (%)

Date	Subtype	*N* = 30	%
Age	⌀ ⌀ Female ⌀ Male	66 71 65	
Gender	Female Male	6 24	20.0 80.0
Nicotine (including former nicotine abuse)	Yes: No: unknown	23 6 1	76.7 20.0 3.3
Disease related Data			
Tumour stage (UICC-classification)	I II III IV	5 8 8 9	16.7 26.7 26.7 30.0
TNM stage	T1 T2 T3 T4 N0 N1 N2 N3 M0 M1	5 8 8 9 22 3 4 1 30 0	16.7 26.7 26.7 30.0 73.3 10.0 13.3 3.3 100.0 0.0
Adjuvant therapy	No adjuvant therapy Radiotherapy Radiochemotherapy	13 12 4	44.8 41.4 13.8

**Fig. 2:**
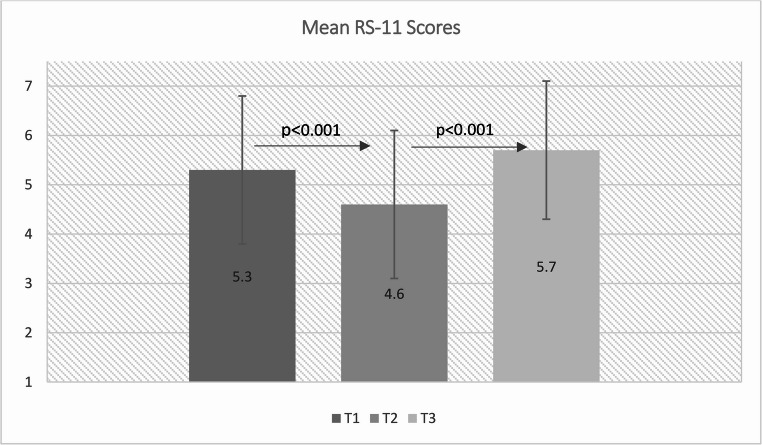
Mean RS-11 score at the different time points T1, T2, and T3. There is a significant reduction from T1 to T2; from T2 to T3 there is a significant increase

Figure 2 illustrates the individual patient resilience scores at the different time points (T1, T2, T3). From T1 to T2, a significant reduction in the RS-11 score was observed (T1: 5.3 (SD 1.5), T2: 4.6 (SD 1.7), *p* < 0.001). From T2 to T3, the score increased again significantly (T2: 4.6 (SD 1.7), T3: 5.7 (1.4), *p* < 0.001).

As presented in Table 2 three patients received regular psycho-oncological support at T3 (T1: *n* = 0; T2: *n* = 23). Seven patients reported regular participation in sports programs at T3 (T1: *n* = 2; T2: *n* = 21). Three patients received also speech therapy (T1: *n* = 1; T2: *n* = 17).

**Table 2:** Utilization of supportive therapeutic interventions at the respective time points (Stated in numbers and percentages). Irregular participation in supportive therapy measures is shown in brackets

Supportive therapeutic measures	T1 *n* = x	%	T2 *n* = x	%	T3 *n* = x	%
Sports activities	2 (2)	6.7 (6.7)	21	70.0	7 (1)	25.9 (3.7)
Psycho-oncology	0	0.0	23	76.7	3 (1)	11.1 (3.7)
Speech therapy	1	3.3	17	56.7	3	11.1
Self-helping groups	0	0.0	(1)	(3.3)	2 (2)	7.4 (7.4)

Table 3 reveals that compared to the preoperative baseline, there was a statistically significant decrease in satisfaction in the domains of physical activity (T1: *p* = 0.01; T2: *p* = 0.53) and enjoyment of food (T1: *p* = 0.01; T2: *p* < 0.001) at T3.

**Table 3:** Satisfaction in various areas of life at points T1, T2 and T3. The content of the nine items is based on Klaus Grawe’s four basic psychological needs (attachment, autonomy, self-esteem enhancement and pleasure seeking)

Satisfaction Items	T1	T2	T3	*P*-value T1/T2	*P*-value T1/T3
Family/Friends	1.4	1.7	1.1	0.13	0.10
Sports activities	2.4	2.9	3.2	0.05	**0.01**
Enjoyable Eating	2.2	3.8	3.2	**< 0.001**	**0.01**
Culture participation	2.6	2.8	3.0	0.38	0.06
Professional perspectives	2.2	2.5	2.5	0.20	0.62
Social recognition	2.1	2.7	2.4	**0.01**	0.15
Sexuality	2.5	2.9	2.2	0.10	0.49
External appearance	2.3	3.2	2.4	**0.01**	0.44
Independence	1.7	3.8	2.1	**< 0.001**	0.10

As part of the outlook for developing a prehabilitation concept, the interval between confirmed diagnosis (histology) and the date of surgery was analysed. This interval averaged 21 days (minimum 9 days, maximum 35 days).

## Discussion

In the present study, the frequency and extent to which patients with oral cavity carcinoma utilized supportive therapeutic interventions during the pre-, peri-, and postoperative phases were examined. In addition, changes in patient-specific resilience and satisfaction across defined areas of life were analysed over the study period. The findings indicate that supportive measures were used considerably more often during the inpatient stay than after discharge. Four months after surgery, only a small proportion of patients reported continuing the initiated interventions on a regular basis. Beyond therapeutic services, individual resilience represents a key factor in coping with stressful life circumstances such as a cancer diagnosis [15]. Patients with higher resilience are better able to cope with stressors, which can have a positive impact on their psychological stability and quality of life (9). In contrast, depressive symptoms may reduce quality of life and are associated with poorer prognosis in head and neck cancer [16]. Factors such as independence, social participation, and body image are closely linked to psychological processing. Family support and social resources may also buffer postoperative stress and promote recovery [17].

In the present study, a significant decline in preoperatively measured resilience was observed immediately after surgery. This decline normalized again four months later. The analysis of individual life satisfaction showed a similar pattern, although with notable limitations.

Considering the existing literature on this topic, previous studies have shown that patients often experience an initial decline in quality of life following surgical treatment of head and neck tumours, which stabilizes over time in a subset of individuals [2, 4]. A relationship between higher resilience and better quality of life has also been reported repeatedly [5, 18]. Thus, the temporal pattern observed in the data collected in this study is consistent with findings from other longitudinal investigations ([4, 5].

Moreover, recent studies suggest that resilience within this patient population is not uniformly expressed. Different resilience types show significant differences in quality of life and psychosocial stability [19]. Social support and socioeconomic factors also appear to play an important role in shaping resilience, particularly during the outpatient phase following surgery [20].

Similar associations have also been described for other tumour entities. Studies in women with breast cancer have shown that high resilience is associated with improved quality of life and enhanced post-traumatic growth [21]. In patients with colorectal cancer, resilience has been found to be closely linked to lower psychological distress and better health-related quality of life [22]. Investigations involving patients with various types of cancer likewise demonstrate that resilience exerts a positive influence on quality of life and coping with the disease [23]. These findings underscore that resilience, regardless of tumour type, represents a key factor in oncological care.

Against this background, the early integration of supportive care is of particular importance. In this study, many patients utilized supportive therapies such as exercise programs or speech therapy immediately after surgery, but participation declined significantly over time. Psycho-oncological support was used by only a small number of patients during the outpatient phase. Similar gaps in care after hospital discharge have been reported in other studies [24, 25]. The literature cites potential reasons such as a lack of follow-up services, organizational barriers, or insufficient coordination between inpatient and outpatient care. Patient-related factors—including limited mobility or lack of motivation—may also play a role. Comparable observations have been reported repeatedly [3, 24, 25].

Changes in eating habits and lifestyle following treatment for head and neck cancer often lead to withdrawal from social activities [24]. Early rehabilitation measures can help minimize functional impairments [25]. These findings are consistent with the present study, which shows a marked reduction in satisfaction regarding physical activity and eating behaviour [3].

Compared with other studies, notable differences emerged particularly in the extent and dynamics of the observed changes. While some investigations describe a more prolonged reduction in quality of life [3, 4], the present study found partial improvement after only four months in the areas of independence, physical appearance, and social recognition. This development may be attributable to individual adjustment processes and rehabilitative progress, as suggested in parts of the literature [5].

The observed changes in life satisfaction illustrate that postoperative limitations extend well beyond the inpatient phase. The most pronounced reductions in satisfaction four months after surgery occurred in the domains of physical activity and enjoyable eating. This highlights the substantial impact that functional impairments—such as swallowing difficulties or mobility issues—can have on quality of life. These observations align with the findings of comparable studies [3, 5]). Surgical interventions frequently lead to initial psychosocial impairments, yet a proportion of patients experience stabilization within several months [5]. Patients also tend to develop new coping strategies to better compensate for functional limitations [3]. Furthermore, the identification of distinct resilience profiles in more recent work supports this interpretation [19].

The results of this study suggest that the early postoperative period represents a critical window for specifically addressing functional and psychosocial impairments. A structured and consistent use of supportive measures after hospital discharge may help prevent long-term limitations and stabilize quality of life. This is in line with the recommendations of the WHO and MASCC, which define supportive care as an integral component of oncological treatment [6, 7].

Clear transition structures, standardized follow-up plans, and improved coordination between inpatient and outpatient care are essential for this purpose. Multimodal programs that combine physical, psycho-oncological, and social components appear particularly suitable to meet these needs.

Another area to which our findings provide insight is prehabilitation. Numerous studies across various tumour entities have described the potential of preoperative preparation to improve patient outcomes [26–29]. Our findings confirm that the time intervals between diagnostic confirmation and definitive tumour surgery vary considerably, highlighting the importance of structured preoperative planning. Particularly noteworthy is the increasing relevance of perioperative use of pembrolizumab in patients with advanced squamous cell carcinoma [30]. Necessary structural adjustments must be explicitly addressed in the development of suitable prehabilitation programs.

### Limitations

The present study has several limitations that must be considered when interpreting the results. A key limitation is the relatively small sample size of 30 patients, which may restrict statistical power and limit the generalizability of the findings. Moreover, the study employed a monocentric design, making it impossible to exclude site-specific characteristics. Another limiting factor is the observation period, which was restricted to four months; long-term developments could therefore not be assessed. In addition, only patients who underwent surgical treatment were included, meaning that other therapeutic modalities were not represented. It should also be noted that the data collection was carried out by a doctoral student under the supervision of the study director, which means that there is a risk of various biases. These include, in particular, interviewer bias, i.e., the unconscious influencing of patients’ answers by the tone of voice and facial expressions of the interviewer; social desirability bias, i.e., the giving of socially desirable answers; and distortions in sensitive areas [31–33]. We attempted to reduce this by using clinically validated questionnaires (RS-11 score) and standardised questions.

All things considered, it has to be said that further multicentre studies with larger patient cohorts and longer follow-up periods would be beneficial to confirm and expand upon these findings. This would also open up the possibility of differentiating between different tumour stages when using supportive care, thereby supporting the development of individual treatment concepts.

## Conclusion

The present study provides an overview of the use of supportive therapeutic interventions in patients with oral cavity carcinoma at a German university hospital at three distinct time points. Notably, while supportive therapies are well established during the inpatient period, they are often not consistently continued after discharge. Since, in addition to functional rehabilitation, psychosocial factors such as individual patient resilience and social support demonstrably play a crucial role in the recovery from malignant head and neck tumours, outpatient follow-up care should be organized in a more structured and patient-centered manner. There is also considerable potential for prehabilitation strategies. When designing new concepts, the integration of cutting-edge therapeutic approaches—such as perioperative immunotherapy—is essential and forms a fundamental pillar for quality-enhancing care structures.

## Data Availability

No datasets were generated or analysed during the current study.
